# The imbalance of self-reported wanting and liking is associated with the degree of attentional bias toward smoking-related stimuli in low nicotine dependence smokers

**DOI:** 10.3389/fpsyg.2024.1356434

**Published:** 2024-05-14

**Authors:** Domonkos File, Bela Petro, Petia Kojouharova, Lili Kővári, Zsófia Anna Gaál, Zsolt Demetrovics, István Czigler

**Affiliations:** ^1^Institute of Psychology, ELTE Eötvös Loránd University, Budapest, Hungary; ^2^Institute of Cognitive Neuroscience and Psychology, HUN-REN Research Centre for Natural Sciences, Budapest, Hungary; ^3^Institute of Psychology, Pázmány Péter Catholic University, Budapest, Hungary; ^4^Center of Excellence in Responsible Gaming, University of Gibraltar, Gibraltar, Gibraltar; ^5^College of Education, Psychology and Social Work, Flinders University, Adelaide, SA, Australia

**Keywords:** flicker paradigm, attentional bias, nicotine dependence, incentive sensitization theory, addictive behaviors

## Abstract

**Background and aims:**

The Incentive Sensitization Theory (IST) offers a comprehensive framework that explains how attentional mechanisms contribute to the maintenance and relapse of addictive behavior. However, the extent to which the underlying neuropsychological mechanisms are consciously accessible for report remains unknown. Thus, the aim of this study was to investigate the association between self-reported wanting and liking among smokers and its relationship with detecting changes in smoking-related stimuli.

**Design:**

An online experiment was designed deploying a flicker paradigm with neutral and smoking-related changes, completed by 422 individuals (mean age = 29.1 years, 214 non-smokers, 123 current smokers, and 85 former smokers). Additionally, the Fagerström Test for Cigarette Dependence and the Imaginative Wanting and Liking Questionnaire were administered.

**Findings:**

Consistent with prior research findings, smokers exhibited faster detection of smoking-related changes compared to non-smokers, while former smokers displayed an intermediate level of attentional bias, falling between the levels observed in smokers and non-smokers. Further, higher levels of nicotine dependence were associated with a greater discrepancy between self-reported wanting and liking, which was associated with better change detection performance for high salience smoking-related stimuli in smokers.

**Conclusion:**

These findings support the predictions of IST and support the notion that attentional bias might develops early in the course of nicotine addiction. Furthermore, the results indicate that the underlying cognitive mechanisms might be partially within conscious awareness, which opens up potential avenues for research design, treatment, and interventions.

## Introduction

Attentional bias is commonly observed in individuals with addiction, often manifested in heightened sensitivity to cues related to substance use (Field et al., 2014). This heightened sensitivity contributes significantly to a pronounced inclination toward engaging in drug-seeking behaviors (Berridge and Robinson, 2016). The Incentive Sensitization Theory (IST) provides a comprehensive framework elucidating how attentional mechanisms play a pivotal role in sustaining and triggering relapses in addictive behavior (Robinson and Berridge, 2000).

According to IST, distinct neural mechanisms underlie the motivational (“wanting”) and hedonic (“liking”) effects of substance use. The two systems exhibit distinct reactions to repeated substance administration, with tolerance affecting the “liking” (resulting in reduced responsiveness) and sensitization influencing the “wanting” system (leading to increased responsiveness). Consequently, individuals with substance use disorders may experience an increased desire for the substance, even in the absence of an expectation of pleasure from its consumption (Robinson and Berridge, 2001). IST posits that the motivational and hedonic systems operate independently of conscious cognition, and the conscious perception of liking and wanting emerges through the interaction of brain systems distinct from those associated with “wanting” and “liking” (Robinson and Berridge, 2003). This separation is evident from the study of Winkielman et al. (2005), in which the impact of subliminally presented happy versus angry faces on motivation and affect was investigated, revealing that basic affective reactions operated unconsciously and interacted with incentive motivation. Crucially, IST asserts that in typical situations, the conscious experience of liking and wanting arises from the interplay between the distinct brain systems associated with conscious awareness and unconscious affect (Berridge and Winkielman, 2003), thus it does not rule out the possibility of conscious experience—at least in part—after the expression of “wanting” and “liking” (Anselme and Robinson, 2016).

The psychological process underlying “wanting” involves assigning salience to stimuli and their representations. This process is thought to modify both neural and psychological representations of ordinary stimuli, rendering them highly salient and attention-grabbing (Robinson and Berridge, 2000). Numerous studies have provided evidence of attentional sensitization, as demonstrated in various scenarios. For instance, studies have shown that individuals using marijuana (Field, 2005), alcohol (Townshend and Duka, 2001), tobacco (Field et al., 2009), cocaine (Liu et al., 2011), or engaging in internet use (Nikolaidou et al., 2019) exhibit facilitated information processing for visual stimuli associated with their respective addictions. These studies consistently reveal that addicts, when compared to control subjects, exhibit quicker detection of changes, faster response times, and reduced ability to inhibit distracting visual stimuli when the stimuli are related to their specific addiction.

Given that “wanting” and “liking” are believed to be influenced by subconscious mechanisms, it is crucial to explore the feasibility of assessing them through self-report methods. However, this presents a significant challenge, as obtaining a direct measure of both explicit and implicit aspects of “wanting” and “liking” proves to be difficult, due to limitations in the original research paradigm used in animal studies for investigating these processes in human subjects (see Pool et al., 2016). It is important to emphasize that while the IST suggests that the motivational and hedonic systems operate independently of conscious cognition, it does not rule out the possibility of conscious experience after their expression. Instead, cognitive self-control often contends with “wanting” implying that it may be influenced, at least in part, by conscious cognitive processes (Anselme and Robinson, 2016).

Despite the methodological concerns, a few survey methods were developed, such as the Sensitivity to Reinforcement of Addictive and other Primary Rewards (STRAPR, Goldstein et al., 2010), the Desires for Alcohol (DAQ) (Love et al., 1998) and Speed Questionnaire (DSQ, James et al., 2004; Willner et al., 2005), and the Imaginative Wanting and Liking Questionnaire (File et al., 2022). While these survey methods have yielded findings that are partially consistent with the predictions of IST, their relationship to implicit measures is not clear. The question of the accessibility of “wanting” and liking to the conscious mind holds a dual significance. Firstly, it is of theoretical importance as it sheds light on the degree to which these psychological processes can be consciously experienced. This understanding is crucial for developing therapeutic and intervention approaches aimed at addressing addictive behaviors or other related conditions. Secondly, from a research methodology standpoint, exploring explicit measures (self-reports, conscious evaluations) that are related to behavioral measures justifies the use of large-scale survey approaches, which allows the comprehensive investigation of the associations between wanting and liking and other psychological properties.

The aim of the current study was to investigate the relationship between smokers’ attentional bias toward smoking-related stimuli and self-reported imbalance of the motivational and hedonic systems (wanting minus liking of smoking: WmL), measured with the Imaginative Wanting and Liking Questionnaire. To this end, an online experiment was designed deploying a flicker paradigm (see Rensink et al., 1997). The flicker paradigm is a frequently employed technique in studies investigating change blindness, which refers to the perceptual phenomenon where an observer fails to notice a change in a visual stimulus (Rensink et al., 1997). The flicker paradigm involves a task where individuals are asked to identify a single difference between two otherwise identical visual scenes displayed on a screen. The scenes are presented repeatedly, with a mask in between, until the change is detected (Jones et al., 2006). Interestingly, it takes a surprisingly long time to notice the change, and this process is strongly influenced by attention (Turatto et al., 2003). One reason for directing attention to the changing element is that viewers have a personal interest in the object that carries the change (Rensink et al., 1997), which makes it suitable to study attentional bias toward specific objects, such as those related to smoking, as in the current study. Three conditions were employed, with neutral and smoking-related changes presented for current-, former, and non-smoker participants. Based on File et al. (2022, 2023), a positive correlation was hypothesized between nicotine dependence and WmL (H1) and a positive correlation between WmL and the performance (number of alternations needed for change detection) of smoking-related change detection compared to neutral changes (H2). Also, a positive correlation was hypothesized between nicotine dependence and performance of smoking-related change detection compared to neutral changes (H3).

Based on previous studies (e.g., Jones et al., 2003, 2006), a replication hypothesis was formed, by which a significant difference between smokers, non-smokers, and former smokers in change detection performance was expected in smoking-related changes relative to neutral changes (smokers > former smokers > non-smokers) (H4). Lastly, a significant difference between smokers, non-smokers, and former smokers in change detection performance was expected in case of neutral changes when a smoking related object was present (smokers < former smokers < non-smokers) (H5).

## Methods

### Participants

An online experiment was conducted from February to March 2023. The experiment was advertised as a research project focused on change detection abilities and was distributed through Hungarian language Facebook advertisements without a specific geographical target. The only criterion specified for the target group was that individuals must be 18 years of age or older. Participant recruitment involved implementing a reward system to encourage participation. As an incentive, participants were eligible for a drawing for 10 coupons. The coupons, valued at ~$30 each, could be redeemed at a popular local retail store. Participants were required to provide written informed consent and were guaranteed anonymity. The study adhered to the principles outlined in the Declaration of Helsinki and obtained approval from the United Ethical Review Committee for Research in Psychology of HUN-REN Research Center for Natural Sciences (Reference number 2023-09, received on 6th February 2023). Data were collected by using a secure online platform called Labvenced (see Finger et al., 2017).

Out of the 1,086 participants who started the experiment, 664 (61.14%) did not complete it. The remaining 422 participants who completed the experiment included 295 women (69.9%). Their ages ranged from 18 to 78 years, with a mean age of 29.1 years (SD = 11.15). In terms of education, less than 7.5% had a maximum primary education, 20.3% reported having a vocational degree, 44.3% held a high-school degree, and 27.7% had a college or university degree. Regarding relationship status, 39.8% were single, 57.8% were in some form of romantic relationship (either being in a romantic relationship or married), and 2.4% selected the “other” option.

### Measures

#### Change detection

**Figure 1:**
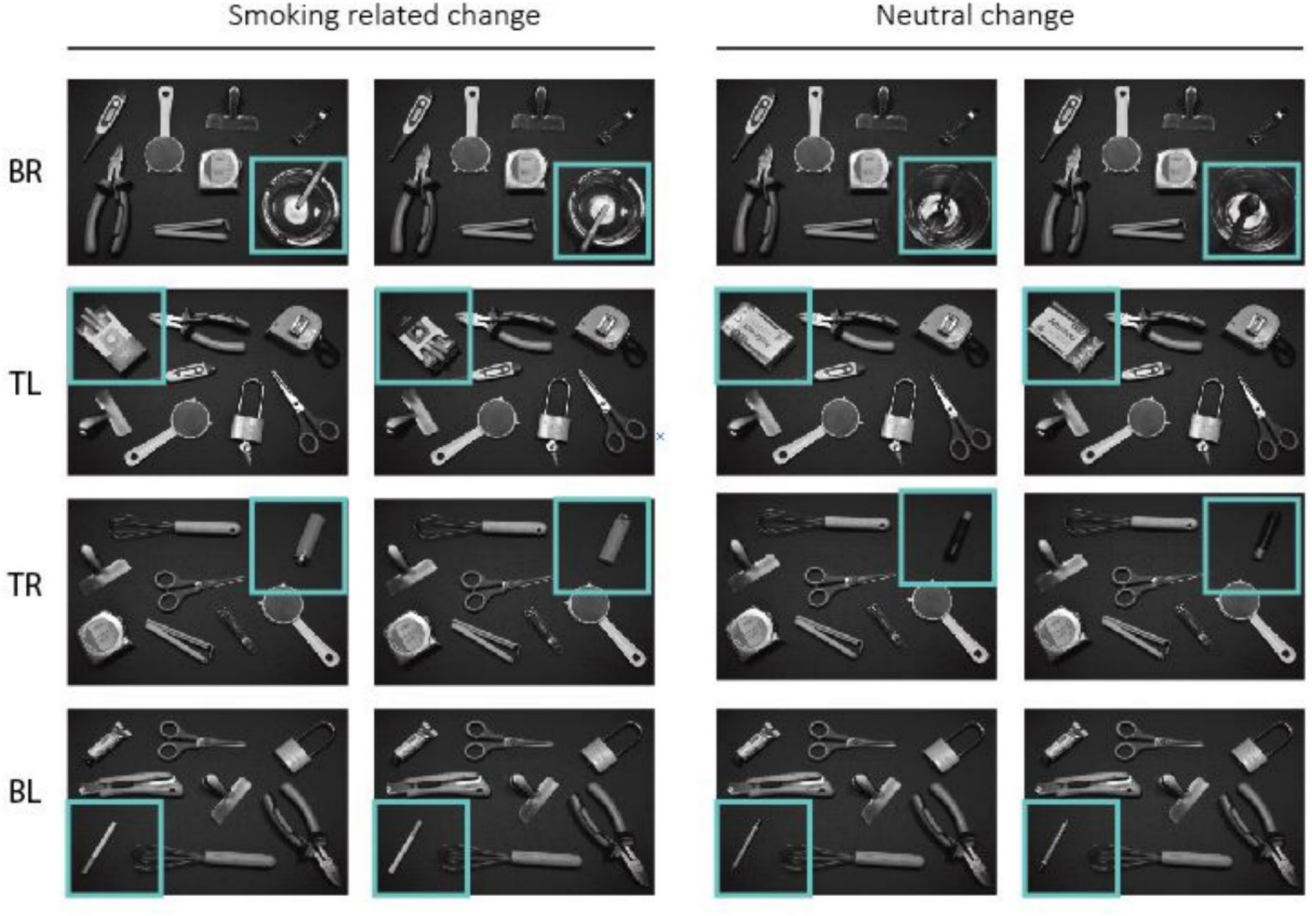
The original stimuli (first column) and the changed stimuli (second column) used in the flicker paradigm in conditions smoking related and neutral change. BR, Bottom-right; TL, Top-left; TR, Top-right; and BL, Bottom-left.

An online flicker paradigm, accessed through web browsers, was employed in the study. The change detection paradigm utilized in this study was based on the experiment conducted by Rensink et al. (1997). The experimental procedure involved the sequential presentation of stimuli as follows: an originating stimulus was displayed for 200 ms, followed by a 500 ms mask, then the changed stimulus for 200 ms, and finally another 500 ms mask. Participants were instructed to identify the change as fast as possible and once detected, click on the changed object. This four-presentation cycle was seamlessly repeated until change was detected and the changed object was clicked. No response was triggered by clicking on an incorrect object (one that had not undergone a change), and the presentation cycle continued. If change was detected, a cross appeared at the center of the screen, and participants were asked to hover the cursor above the cross, which triggered the next block. The experiment consisted of three conditions; Neutral Change (NC), Smoking Change (SC), and Neutral Change Smoking Present (NC-S). In condition NC, all eight objects were household objects and one of them changed. In condition SC, there were seven household objects and one smoking-related (which changed). In the NC-S condition, there were seven household objects (from which one changed) and a smoking-related one (which did not change in this condition). Change was always the 180° rotation of one of the objects. Stimuli were greyscale photographs of eight objects from a top view. There were four locations of interest; the top left and right corner (TL and TR), and the bottom left and right corner (BL and BR). The choice of utilizing the four corners of the screen was motivated by their equal distance from the center of the screen. This decision aimed to ensure that variations in detection performance could not be ascribed to differences in the distance from the initial cursor starting location. Also, contextual objects were positioned in other locations, such as the middle, top middle, and bottom middle. As a result, the specific position of the change within the stimuli was unpredictable. Objects at locations of interest were selected so that their physical properties (e.g., color, height, width, and shape) were generally similar between NC and SC conditions (see Figure 1). There were six blocks in each condition (with four changing objects at the locations of interests and two contextual), thus, in total, 18 blocks were presented in a random order.

The dependent variable of primary interest was the difference in the number of transitions (referred as latency) between NC and SC conditions (NC minus SC). The decision to implement the NC-S condition was driven by two main rationales. Firstly, it aimed to prevent participants from predicting the occurrence of a change specifically related to smoking. By maintaining smoking-related objects without alteration, we aimed to reduce the likelihood of participants anticipating a change in that particular context. Secondly, the inclusion of the NC-S condition was exploratory in nature, aiming to investigate whether the presence of unchanged smoking-related objects would have any potential distracting effects.

#### Cigarette dependence

Nicotine dependence levels were evaluated utilizing the Fagerström Test for Cigarette Dependence (Fagerström, 2011), a tool consisting of six items that assess factors such as cigarette consumption, compulsion to use, and overall dependence. Scores on this test range from 0 to 10, with higher scores indicating a higher degree of physical dependence on nicotine. Furthermore, years of abstinence of former smoker participants and average number of cigarettes per day of current smokers were also assessed.

#### Wanting and liking

In File et al. (2022), the Imaginative Wanting and Liking Questionnaire was developed to measure the imaginative wanting and liking of substance or behavior-related cues. The questionnaire was designed based on the premise that the contemplation of reward cues could elicit measurable wanting in the absence of actual stimuli, as proposed by Berridge et al. (2009). This questionnaire comprises micro-scenarios prompting participants to envision themselves in situations related to substance or behavior use. In this specific study, participants were instructed to imagine holding a cigarette in the right time and place. Subsequent to the imagery prompt, participants were required to report their expected emotions using a ruler spanning from −100 (very bad) to 100 (very good) before, during, and after the cigarette, with three items: (1) How would you feel right before you light the cigarette?, (2) How would you feel during smoking?, and (3) How would you feel after you finished the cigarette?. Furthermore, participants were requested to estimate the amount of willpower required to resist or stop engaging in smoking before, during, and after smoking. This assessment was conducted using a scale ranging from 0 (nothing) to 100 (enormous), encompassing the following items. (1) How much willpower would you need not to light the cigarette and to not smoke in the next 24 h?, (2) How much willpower would you require to resist finishing the cigarette after taking a few puffs and to not smoke in the next 24 h?, and (3) How much willpower would you need in order to not smoke in the next 24 h after you finished the cigarette?.

In assessing the internal consistency reliability of the scale, Cronbach’s alpha was calculated for the wanting and liking items separately. The obtained Cronbach’s alpha value for wanting was 0.961 [95%CI: 0.945, 0.973], indicating high internal consistency among the scale items. The obtained Cronbach’s alpha value for liking was 0.736 [95%CI: 0.603, 0.830], indicating acceptable internal consistency among the scale items. The WmL score was derived by computing the difference between the sum of wanting items (wanting_before + wanting_during + wanting_after) and the sum of liking items (liking_before + liking_during + liking_after).

#### Missing data

While testing WmL related hypothesis (H1–H4), missing data of WmL variables (11.6%) were replaced with median imputation. This choice was motivated by the non-normal distribution of WmL variables, and the use of median imputation was preferred as it preserves the variable’s overall distribution, maintaining validity comparable to more intricate methods for managing missing values (Berkelmans et al., 2022).

### Analyses

Advantage in change detection performance for smoking-related changes (smoking related attentional bias) was formed as the difference in alternations to detect change between NC and SC conditions. The Shapiro–Wilk normality test was conducted to assess normality, while the Levene test was employed to evaluate the homogeneity of variances between groups.

Since the assumptions of normality were violated in case of Hypotheses 1–3, Spearman’s rank-order correlations were calculated. Since the assumptions of normality were violated, Kruskal-Wallis test was utilized for the group comparison in case of Hypotheses 4–5.

Upon initial analysis of WmL-related hypotheses (H1–H4), we observed non-significant results when including all four smoking-related changes in our study (see Appendix 1). Given the low average nicotine dependence score of our sample (2.13, SD = 2.38), a potential limitation arose regarding the presence of attentional bias in the low salience changes. To address this concern, we conducted a comparison of average latencies for the four smoking-related changes using a Kruskal-Wallis test. The results revealed significant differences between the four changes [χ^2^ (3, *N* = 344) = 21.03, *p* < 0.001]. Subsequently, pairwise comparisons were performed using Dunn’s test, which indicated that the detection of change was significantly faster in locations BR (cigarette in ashtray) and TL (pack of cigarettes) compared to locations BL (cigarette) and TL (lighter). Detailed information on the average latencies can be found in Appendix 2, and the *p* values of Dunn’s test are available in Appendix 3. Consequently, while testing the WmL-related hypotheses (H1–H4), we included only the two most salient stimuli (BR and TL).

Statistical analyses were carried out in R (4.0.2) and CogStat (Krajcsi, 2020).

## Results

Out of the 422 participants, 214 were non-smokers (mean age: 26.68, SD = 10.34, 70.1% female), 123 current smoker (mean age: 28.70, SD = 10.70, 67.5% females, average Fagerström score: 3.65, SD = 2.4), and 85 former smoker (mean age: 30.72, SD = 12.71, 72.9% females, average Fagerström score: 2.99, SD = 2.52). For an overview of the variables used in the analyses, see Table 1.

**Table 1:** Overview of the variables used in the study.

	Smoker	Former-smoker	Non-smoker
*N*	123	85	214
Mean age (SD)	28.70 (10.70)	30.72 (12.71)	26.68 (10.34)
Females (%)	67.5%	72.9%	70.1%
Neutral Change (SD)^*^	6.63 (1.63)	7.01 (2.2)	6.72 (2.58)
Smoking Change (SD)^*^	6.19 (1.97)	6.21 (1.74)	6.41 (2.02)
Neutral-change smoking present (SD)^*^	6.42 (1.63)	6.89 (2.32)	6.44 (1.69)
Change detection performance difference between NC and SC conditions (SD)^*^	0.42 (2.02)	0.82 (2.47)	0.31 (2.35)
Cigarette dependence (Fagerström score) (SD)	3.65 (2.4)	2.88 (2.52)	-
WmL (SD)	12.17 (11.75)	2.98 (7.96)	-

^*^Mean number of presentation cycles to detect change.

There was a weak positive correlation between nicotine dependence and WmL [H1, *r_s_*(121) = 0.28, *p* = 0.002]. Also, there was a moderate positive correlation between WmL and change detection performance of smoking related changes [H2, *r_s_*(121) = 0.33, *p* < 0.001]. Contrary to our expectations, the correlation between nicotine dependence and change detection performance of smoking related changes was not significant [H3, *r_s_*(121) = 0.07, *p* = 0.456]. For an overview, see Table 2.

**Table 2:** Spearman’s rank-order correlation coefficients between variables, nicotine dependence, WmL, and change detection performance of smoking related changes.

	1.	2.
1.Change detection performance		
2.Nicotine dependence	0.07 [−0.11, 0.24], *p* = 0.456	
3.WmL	0.33 [0.16, 0.48], *p* < 0.001	0.28 [0.11, 0.43], *p* = 0.002

**Figure 2:**
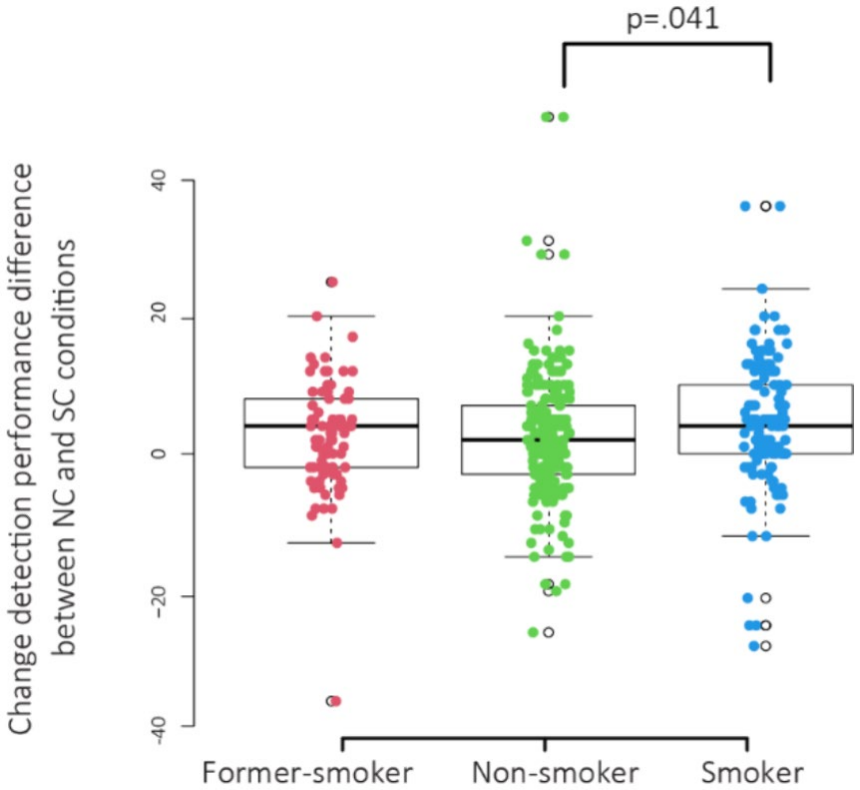
Boxplots of change detection performance difference between NC and SC conditions (mean number of “flickers”) across groups former-smokers, non-smokers, and smokers.

Since the smoking related attentional bias variable within the Smoker group did not follow a normal distribution, Kruskal-Wallis test was used to compare groups non-smoker, smoker and former smoker (H4). The analysis revealed a significant effect of smoking related attentional bias χ^2^(2, *N* = 422) = 6.37, *p* = 0.041 (Figure 2). The effect size, Omega-squared was small (ω^2^ = 0.00431). *Post-hoc* tests using Dunn’s test indicated that smokers needed fewer stimulus presentations to detect smoking related changes (relative to neutral changes) (*p* = 0.012) than non-smokers. There were no significant differences observed between former smokers and both smokers (*p* = 0.205) and non-smokers (*p* = 0.0.406). There was no significant difference between groups in the Latency of change detection of neutral changes where smoking related object was present relative to neutral changes where no smoking related object was present (NC minus NC-S) [χ^2^(2, *N* = 422) = 0.93, *p* = 0.628] (H5).

## Discussion

The Incentive Sensitization Theory of addictions (Robinson and Berridge, 1993) offers a comprehensive neuropsychological framework to explain attentional sensitization in addictions, which is supposed to be the result of largely implicit cognitive mechanisms. However, the extent to which these underlying mechanisms are consciously accessible for reports remains limited in our current knowledge and is a subject of ongoing debate. For the best of the authors’ knowledge, no study has investigated the link between the imbalance of self-reported wanting and liking and attentional bias for addiction related stimuli. Thus, the main objective of this study was to investigate the association between self-reported wanting and liking measured with the Imaginative Wanting and Liking Questionnaire of smokers and their performance in detecting changes in smoking-related stimuli on a relatively large sample.

In line with previous research (e.g., File et al., 2022, 2023), the current study found a positive relationship between nicotine dependence and the self-reported imbalance of wanting and liking. Specifically, higher levels of nicotine dependence were associated with a greater discrepancy between wanting and liking, indicating a stronger desire for nicotine relative to the pleasurable experience derived from it.

The primary discovery of the study highlighted a positive correlation between wanting-minus-liking (WmL) and change detection performance in smoking-related stimuli among smokers, aligning with the predictions of IST (Robinson and Berridge, 1993). This correlation between the self-report measure of IST and attentional bias lends support to the credibility of self-report assessments, indicating their ability to capture not only conscious evaluations but also their connection to relevant cognitive processes. Considering that the original research paradigm is not suitable to investigate wanting and liking in humans (Tibboel et al., 2011), it becomes imperative to undertake a thorough exploration of diverse approaches. It is unquestionable that physiological and neurobiological measures are better suited for assessing IST, as evident in the methods commonly employed in the realm of human IST studies (Pool et al., 2016). However, the current results indicate that self-report methods are potentially useful too, which opens avenue for survey studies, allowing researchers to explore a more comprehensive understanding of psychological correlations of IST. In line with that, WmL demonstrated heightened sensitivity to attentional sensitization compared to the addiction score itself, suggesting that its utilization can potentially contribute to a more comprehensive understanding of addictive behaviors. As Field and Cox (2008) summarized, it is not clear that the strength of attentional bias is dependent on the severity of nicotine dependence. Previous studies reported positive correlation (e.g., Zack et al., 2001), negative correlation (Mogg et al., 2005), or no relationship (Munafò et al., 2003). The current results showed no correlation between attentional bias strength and nicotine dependence, but there was a positive correlation between attentional bias strength and WmL. A possible explanation for the inconsistent results might be that nicotine addiction is usually measured with the Fagerström Test for Nicotine Dependence (Heatherton et al., 1991), which shows floor effects when measuring populations of light smokers (Etter et al., 1999). Another potential explanation is that the attention-capturing capability of substance-related stimuli is not directly linked to dependence severity (as in Ryan, 2002 or Jones et al., 2006), but is more directly associated with the imbalance between wanting and liking. In line with that, Hobson et al. (2013) reported an association between change detection performance with levels of craving, but not with alcohol consumption. However, as both of these explanations remain speculative, additional research is required to explain the inconsistent results from previous studies.

Previous research has pointed out that attentional bias may play a role in sustaining smoking habits and contributing to relapse (Masiero et al., 2019). However, the current findings suggest that the observed attentional bias likely emerges early in the progression of problematic use, given the relatively low level of nicotine dependence in the sample, as indicated by an average Fagerström score of 3.65 (SD = 2.4), indicating low to moderate dependence. This observation is consistent with Jones and Bruce’s (2005) proposal of a graded continuity of attentional bias across the consumption continuum. This implies that altered attentional processes may not only be confined to maintaining smoking and contributing to relapse but also extend to the development of addiction by emphasizing smoking-related environmental cues that trigger the behavior.

While the majority of studies have concentrated on the association between the degree of addiction and the temporal aspects of attentional bias, such as the time needed to detect changes (Jones and Bruce, 2005) or the speed differences between congruent and incongruent conditions in Go/no-Go or Stroop tasks (Zack et al., 2001; Munafò et al., 2003), the present findings suggest that exploring alterations in the set of cues capable of triggering attentional bias could be a promising avenue for research. In the current study, only the most salient, closely tobacco-related stimuli (pack of cigarette and cigarette on ashtray) were linked to WmL, although the sensitized attentional system is assumed to be not restricted to direct substance cues, other associated cues are able to activate it (Robinson and Berridge, 1993). The restricted generalization observed in the current study may be attributed to the relatively low level of nicotine dependence of the sample. To draw more robust conclusions, future studies should consider a more diverse set of stimuli and include participants with higher levels of nicotine dependence. In the present study, we successfully replicated a widely observed group difference (e.g., Jones et al., 2003, 2006); smokers exhibited faster detection of smoking-related changes compared to non-smokers, indicating an attentional bias toward smoking-related stimuli. The practical significance of the observed bias is heightened by the fact that the study was conducted outside the controlled confines of a laboratory setting, taking place within a web browser under uncontrolled conditions. The current results only partially supported the notion that the altered sensitivity of the attentional system persists beyond the cessation of actual smoking, as proposed by Berridge et al. (2009). Former smokers displayed an intermediate level of attentional bias, falling between the levels observed in smokers and non-smokers. Notably, the attentional bias in former smokers did not significantly differ from either group.

Overall, the findings of the current study support the predictions of the Incentive Sensitization Theory (IST) and contribute to the growing literature on that attentional mechanisms play a crucial role in addictive behaviors. The results suggest that self-reported wanting and liking may be linked to the implicit cognitive mechanisms described in IST, which suggests that using self-report measures in the field of IST should not be discarded at this early phase of research. Instead, developing and testing such measures can potentially provide valuable information complementing experimental data. Moreover, the possibility that the dynamics of wanting and liking are at least partially accessible to conscious awareness opens up possibilities for treatment and interventions. For instance, attention bias modification induces enduring automatic and sustained avoidance responses to cues associated with smoking, making it a potential strategy to help smokers quit (Lopes et al., 2014). In line with that, File et al. (2023) reported that self-reported wanting showed a positive correlation with intention to quit smoking, indicating that by directing attention to the motivational and hedonic aspects of smoking, it may be feasible to enhance motivation to quit.

### Limitations

A few limitations warrant consideration. First, the cross-sectional design employed in this study limits our ability to infer causation or assess temporal relationships between variables, precluding the examination of dynamic changes in the course of development of nicotine addiction. Moreover, although the non-completion rate was notably high at approximately 61%, it aligns with the typical response rate observed in online surveys, as demonstrated by Wu et al. (2022). Furthermore, the study did not document the device and input modality (touch screen, mouse), potentially leading to the introduction of systematic biases into the results. Additionally, to avoid drawing attention to smoking-related stimuli prior to the experiment, the questionnaires were administered afterwards. This approach hindered the analysis of the characteristics of participants who did not complete the study. To enhance the robustness of findings, future studies should consider including subjects with higher nicotine dependence levels to adequately test assumptions related to the impact of attentional bias on cue generalization.

## Data availability statement

The raw data supporting the conclusions of this article will be made available by the authors, without undue reservation.

## Ethics statement

The studies involving humans were approved by United Ethical Review Committee for Research and Psychology. The studies were conducted in accordance with the local legislation and institutional requirements. The participants provided their written informed consent to participate in this study.

## Author contributions

DF: Conceptualization, Data curation, Formal analysis, Investigation, Methodology, Visualization, Writing – original draft, Writing – review & editing. BP: Conceptualization, Writing – original draft, Writing – review & editing. PK: Writing – original draft, Writing – review & editing. LK: Writing – original draft, Writing – review & editing. ZG: Writing – original draft, Writing – review & editing. ZD: Writing – original draft, Writing – review & editing. IC: Conceptualization, Funding acquisition, Supervision, Writing – original draft, Writing – review & editing.

## Appendix

Appendix 1

H2: Testing the correlation between WmL and performance of smoking-related change detection compared to neutral changes (without omitting the two low-salience changes) revealed no significant correlation (*r_s_*(121) = 0.03, 95% CI: [−0.152, 0.202], *p* = 0.774).
H3: Testing the correlation between nicotine dependence and performance of smoking-related change detection compared to neutral changes (without omitting the two low-salience changes) revealed no significant correlation (*r_s_*(121) = 0.02, 95% CI: [−0.155, 0.199], *p* = 0.806).

Appendix 2

Average alternations for non-smokers, smokers and former-smokers for conditions Neutral Change, Smoking Change and Neutral-Change Smoking Present presented to the 4 quadrants. TL: top-left; TR: top-right; BL: bottom-left; BR: bottom-right.

**Table:**

Non-smoker	TL	TR	BL	BR
Neutral Change	6.63 (2.87)	6.48 (3.17)	5.75 (2.36)	5.73 (3.29)
Smoking Change	6.22 (3.13)	6.08 (2.67)	6.74 (3.42)	5.27 (1.97)
Neutral-Change Smoking Present	6.41 (2.86)	6.27 (2.73)	7.0 (3.45)	5.74 (2.41)
Smoker	TL	TR	BL	BR
Neutral Change	6.45 (2.87)	6.96 (3.66)	6.39 (2.87)	6.14 (3.15)
Smoking Change	5.69 (2.23)	6.16 (2.25)	6.6 (3.71)	5.13 (2.37)
Neutral-Change Smoking Present	6.6 (2.88)	6.08 (2.88)	7.71 (4.46)	6.64 (3.66)
Former smoker	TL	TR	BL	BR
Neutral Change	6.74 (2.71)	6.02 (2.51)	6.0 (2.27)	5.49 (2.33)
Smoking Change	5.89 (2.21)	6.04 (2.81)	6.52 (2.84)	5.53 (2.52)
Neutral-Change Smoking Present	6.21 (2.06)	6.37 (2.79)	7.37 (3.99)	5.73 (2.53)

Appendix 3

Comparison of latency values for the four smoking-related changes: P-values of Dunn’s test

**Table:**

	BL	TL	BR	TR
BL	1.000	0.000	0.034	0.197
TL	0.000	1.000	0.020	0.002
BR	0.034	0.020	1.000	0.405
TR	0.197	0.002	0.405	1.000
